# P-1698. Prevalence of Anaerobes, *Enterococcus,* and *Pseudomonas* Among Patients with Complicated Biliary Infections

**DOI:** 10.1093/ofid/ofae631.1864

**Published:** 2025-01-29

**Authors:** Taylor Mitsuuchi, Janice Lau, Mike Hori, Joanne Huang

**Affiliations:** Valley Medical Center, Renton, Washington; Valley Medical Center, Renton, Washington; Valley Medical Center, Department of Infectious Diseases, Renton, Washington; Valley Medical Center, Renton, Washington

## Abstract

**Background:**

Current 2010 Infectious Diseases Society of America (IDSA) guidelines indicate that community acquired biliary infections do not require antimicrobial therapy directed against *Enterococcus* spp., *Pseudomonas* (PSA), or anaerobes in the absence of select risk factors. We evaluated empiric prescribing practices for complicated biliary infections at Valley Medical Center (VMC).

**Methods:**

This was a single center retrospective study including patients 18 years or older with complicated biliary infections admitted at VMC from 1/1/2018 - 2/28/2022. Patients who did not require surgical intervention, had no cultures collected, or noted cephalosporin allergy were excluded. The primary outcome was to evaluate the prevalence of anaerobic, *Enterococcus* spp., and PSA bacteria isolated from culture. Secondary outcomes evaluated rate of Intensive Care Unit (ICU) admissions, all cause 30-day readmission, and *C. difficile* infections (CDI).

**Results:**

191 patients with complicated biliary infections with culture data were included. Anaerobes and *Enterococcus* spp. were the most commonly isolated organisms, accounting for 19.4% and 11.5%, respectively. Of anaerobic cultures obtained, 6 (3.1%) were identified as gram-negative anaerobes and 26 (13.6%) were identified as gram-positive anaerobes. *Pseudomonas* spp. was uncommon (0.5%). 78 (40.8%) had negative cultures. 85 (44.5%) received empiric ceftriaxone. Of those on ceftriaxone, 75 also received metronidazole. 88 (46.1%) received empiric piperacillin tazobactam and the remaining 18 patients received alternative therapies. 59 (30.9%) were either admitted or transferred to ICU during their index admission. Of these, 48 (81.4%) were on piperacillin tazobactam. Similar all-cause 30-day readmission was observed among patients on ceftriaxone and piperacillin tazobactam (43.4%). Diagnosis with CDI within 90 days of discharge was uncommon (1.6%).

**Conclusion:**

PSA and Gram-negative anaerobe prevalence was low; *Enterococcus* and Gram-positive anaerobes was higher. Lower rates of ICU admission and CDI were observed in patients on ceftriaxone, though may be in part due to less severe illness. Additional studies are needed to evaluate risk factors that may be predictive of *Enterococcus* and anaerobic infection.

**Disclosures:**

**All Authors**: No reported disclosures

